# Social participation in patients with multiple sclerosis: correlations between disability and economic burden

**DOI:** 10.1186/1471-2377-14-115

**Published:** 2014-05-27

**Authors:** Arnaud Kwiatkowski, Jean-Pierre Marissal, Madani Pouyfaucon, Patrick Vermersch, Patrick Hautecoeur, Benoît Dervaux

**Affiliations:** 1 PRES Lille Nord de France, Lille, France; 2 Department of Neurology, Hôpital Saint Vincent de Paul, Groupement des Hôpitaux de l’Institut Catholique de Lille, Bd de Belfort BP 387, Lille cedex F-59020, France; 3 Department of Public Health, Université Lille Nord de France (EA2694), Lille, France; 4 Faculté Libre de Médecine, Université Catholique de Lille, Lille, France; 5 Faculté Libre des Sciences Economiques et de Gestion, LEM UMR CNRS 8179, Université Catholique de Lille, Lille, France; 6 Department of Medicine, Centre Hospitalier d’Hazebrouck, Hazebrouck, France; 7 Department of Neurology, Université Lille Nord de France (EA2686), Lille, France

**Keywords:** Multiple sclerosis, Social participation, Economic burden, Quality of life, Natalizumab

## Abstract

**Background:**

Economic costs related to treatment of multiple sclerosis (MS) must be justified by health state, quality of life (QOL) and social participation improvement. This study aims to describe correlations between social participation, economic costs, utility and MS-specific QOL in a sample of patients with MS (pwMS).

**Methods:**

We interviewed 42 pwMS receiving natalizumab and collected clinical data, direct medical costs, productivity loss, utility (EQ5D-VAS), MS-specific QOL (SEP-59), social participation with the Impact on Participation and Autonomy questionnaire (IPA). We performed descriptive and correlation analyses.

**Results:**

41 pwMS, with a mean Expanded Disability Status Scale (EDSS) score of 4.0, completed questionnaires. Mean annual global cost per patient was 68448 +/-33374 Euros and increased with EDSS (r = 0.644), utility (r = -0.456) and IPA (r = 0.519-0.671) worsening. Mean utility was 0.52 +/- 0.28. Correlations between IPA and QOL (EQ5D-VAS or SEP-59) were observed (r = -0.53 to -0.78). Association between QOL and EDSS was smaller (EQ5D-VAS) or absent. Productivity losses were poorly correlated to EDSS (r = 0.375).

**Conclusion:**

Moderate to strong correlations of social participation with clinical status (EDSS), QOL, utility and economic costs encourage exploring better these links in larger cohorts. The stronger correlation between social participation and QOL than between EDSS and QOL needs to be confirmed.

## Background

Multiple sclerosis (MS) is a chronic inflammatory, demyelinating and neurodegenerative disease of the central nervous system causing various neurological symptoms, including motor, sensory, visual or cognitive troubles. MS primarily strikes adults between 18 and 45 years and has several forms of presentation [1]. 85% of patients have relapsing-remitting MS (RR-MS) with periods of defined relapse (with either fully recovery or residual deficit after recovery) and periods characterized by a lack of disease progression. Secondary progressive MS is an initial relapsing-remitting course followed by progression with or without occasional relapses (50% of RR-MS). In about 15% of cases, primary progressive MS is a gradual, nearly continuously worsening baseline with no distinct relapse. Consequences of MS progression can be clinically described with different scales or tools, but Expanded Disability Status Scale (EDSS) remains the most used outcome in worldwide neurological community, even if numerous shortcomings have been discussed [2]. Progressively, patients’ physical activities, ability to generate income [3] and social participation [4] can be restricted. From the beginning of the disease, MS significantly alters quality of life (QOL) [5,6].

For over 15 years, clinical trials of various subcutaneously or intramuscularly immunomodulatory drugs have demonstrated a modest reduction in relapse rate and limited effects on disability progression, with a well-known long-term safety but a poor daily tolerability [1]. Recently, second-line treatments such as natalizumab [7] or fingolimod [8,9] showed greater effects on relapse rate and convincingly on disease progression. However serious adverse events and long-term safety remain a concern with such drugs and require closed monitoring survey.

Treatment prices are an important vector of cost in MS, essentially in initial phases of the disease [10] and cost-effectiveness of all these drugs is questionable [11]. Economic evaluation is playing an increasing role in political decisions for resources allocation and health policies. In the current economic context with limited resources, national or international institutions need up-to-date economic burden studies and recommend assessment of patient-reported outcomes (PRO) as utilities in clinical trials [12,13]. Utility is most often assessed by preference-based systems and through generic QOL measurement tools, like EuroQol (EQ5D-VAS), Short Form Health Survey (SF-6D) or Health Utilities Index III (HUI-III). Most studies in MS field have used EQ5D-VAS, in which explored dimensions have important link to mobility capacities [14]. However, several important domains of impairments or limitations, as cognition, vision and others, are poorly taken in count by these tools. Specific QOL scales are available for patients with MS, but these do not allow measuring utilities. Patient’s working capacity is well evaluated with loss of production in global burden studies, but others social disadvantages are only approached with utility scales.

Our hypothesis is that disability in MS and particularly social participation impairment should be better correlated with utility, MS-specific QOL and economic costs rather than health state described by an impairment scale as EDSS. If it was verified, this relationship could lead to an interesting tool for evaluation of health policy. Indeed, a health-promoting action that could correct functioning or social participation deficits could be economically recovered.

Our primary objective was to describe consequences for patients treated by natalizumab in term of disabilities and economic burden due to MS. In this study, disabilities covered impairments, activity limitations and social disadvantages, using EDSS and a measurement of autonomy and social participation. Secondary, we examined correlations between usual tools, as EDSS, MS-specific or generic QOL scales, and the "impact on participation and autonomy questionnaire" (IPA).

## Methods

### Study participants

Researchers recruited consecutively a sample of patients with MS who have monthly infusion of natalizumab, at the Neurology Department, St Vincent de Paul Hospital, Lille, France. Inclusion criteria were a diagnosis of MS [15], an on-going treatment with natalizumab and to be at least 18 years old. Exclusion criteria were inability to read/understand French, or unwillingness to participate. Table 1 provides a summary of participants’ demographic and medical information. This preliminary study was a part of a larger study that was approved by the local ethic committee named "Comité de Protection des Personnes Nord Ouest IV" (number SC 11/02). A written informed consent was obtained from patients who agreed to participate. Due to non-randomised inclusion of participants in a unique centre and to limited time available to this first study, the investigators chose to include only a representative sample of patients who were treated with the same molecule, here natalizumab.

**Table 1:** Demographics, utility, MS-specific QOL, IPA scores and economic costs in entire survey sample and 3 groups separated by EDSS values

	**Survey sample**	**EDSS**		
		**0-3.5**	**4.0-5.5**	**≥6.0**
**No**	41	17	17	7
**Age (year)**	42.8 (9.5)*	37.9 (9.7) §	45.5 (5.4) §	48.3 (12.2)
**Sex-ratio M/W**	7/34	3/14	2/15	2/5
**Education level (year)**	12.0 (3.3)	12.1 (3.4)	12.3 (3.4)	10.9 (3.1)
**Disease duration (year)**	11.7 (7.4)*	8.5 (5.3) §	11.1 (4.8) ¶	20.9 (10) § ¶
**EDSS**	4.0 (1.6)*	2.5 (0.8) §	4.7 (0.6) §	6.2 (0.4) §
**No of currently employed patients**	24	14	10	0
**Full time employment (%)**	39%	53%	15%	0%
**EQ5D-VAS score**	0.52 (0.28)*	0.65 (0.31) §	0.44 (0.23) §	0.42 (0.24)
**MSQOL-P**	51.1 (19.1)	59.5 (21.4)	44.1 (17.0)	47.9 (10.1)
**MSQOL-M**	55.1 (24)	60.2 (23.2)	51.8 (23.8)	45.0 (14.3)
**IPA**				
**Autonomy indoors**	0.91 (0.82)*	0.54 (0.57) §	1.17 (0.83) §	1.34 (1.14)
**Family role**	1.7 (0.8)*	1.21 (0.67) §	2.14 (0.68) §	1.91 (0.75)
**Autonomy outdoors**	1.89 (1.08)*	1.19 (0.88) § ¶	2.49 (0.89) §	2.36 (0.96) ¶
**Social life**	1.1 (0.65)*	0.87 (0.69) §	1.14 (0.54)	1.71 (0.5) §
**Work and education**	2.54 (1.32)*	1.74 (1.18) §	3.22 (1.0) §	3.12 (1.28)
**Costs (€):**				
**Total cost**	68448 (33374)*	52505 (26570) §	70337 (31975)	102581 (26963) §
**Direct medical cost**	34210 (3818)*	33062 (3793) §	34571 (3972)	36123 (2893) §
**Direct non medical cost**	10521 (8297)*	5525 (4651) § ¶	11885 (7289) ¶	19290 (9686) §
**Informal care**	2902 (2689)*	1594 (1637) §	3271 (2285)	5181 (3981) §
**Indirect cost**	23725 (25871)	13918 (20907) §	23879 (27042)	47167 (20799) §

Mean (Standard deviation).

*Significant difference between groups (one-factor ANOVA on ranks), p ≤ 0.05.

§, ¶ Significant difference between two groups (Bonferroni post-hoc test), p ≤ 0.05.

### Instruments

#### Social participation

The Impact on Participation and Autonomy Questionnaire (IPA) assess the perceived personal impact of chronic disability on participation and autonomy. Effectively, World Health Organisation’s International Classification of Functioning, Disability and Health (ICF) was developed in 2001, inspired by theorical researches in the forty last years, such as Amartya Sen’s theory who define notions of functioning and capabilities. In this context, the conceptual framework of social participation has been defined as "the involvement of an individual in life situations in relation to health conditions, body function and structures, activities and contextual factors". The IPA was developed and validated by Cardol and colleagues in the Netherlands [16]. The IPA was translated into English and validated for using by patients which have disabilities due to MS, rheumatoid arthritis and spinal cord injury [17,18]. A French version was equally validated by Canadian researchers for elderly people [19]. The IPA contains 32 items which load onto five domains termed: autonomy indoors; family role; autonomy outdoors; social life and relationships; work and education. Scores for each domain range from 0 ("very good") to 4 ("very poor"); lower scores indicate better social participation and autonomy. In the validation study of IPA questionnaire from UK, the control group participants with mild or no disability had median scores near from 0 (0 to 0.2) in all five domains from IPA. Patients with MS experienced median scores between 0.67 to 2.2 [18].

### Quality of life

#### Generic QOL questionnaire and utility

The EQ5D-VAS is a generic preference-based measure of health-related QOL that consists of two parts. The first part includes five domains (each divided in 3 levels: no problem, some problems and extreme problems), which are termed: mobility, capacity for self-care, conduct of usual activities, pain/discomfort and anxiety/depression. It permits to define 243 health states, which are valued in term of utility using a value set recently published in French population [20], where 0 is equivalent to death and 1 is a good health. The second part is a Visual Analogue Scale to measure self-perceived health with values from 0 to 100 (data not shown).

#### MS specific QOL questionnaire

SEP-59 questionnaire is the validated French version [21] of MSQOL-54, an international-used MS specific QOL questionnaire [22], which combines the MOS SF36 together with MS specific items. It contains 59 items divided in 15 domains named: physical function, role limitation – physical, role limitation – emotional, social function, pain, energy/fatigue, emotional well-being, health perception, health distress, cognitive function, sexual function, sexual satisfaction, overall QOL, sleep and social support. Scores for each domain range from 0 to 100; higher scores indicate better health status, except for the pain subscale. The MSQOL-54 questionnaire, scored according to the User’ Manual, permits to produce two aggregated scores which are a physical health QOL score (MSQOL-P) and a mental health QOL score (MSQOL-M).

#### Medico-economic, demographic and clinical data

The investigators completed a detailed questionnaire for each patient. This included questions on socio demographic characteristics, health status (type of MS, disease duration, relapse rate in the last year), clinical examination with EDSS measurement, concomitant health conditions, questions to determine direct costs (medical drugs, out-patient visits, hospitalizations related to MS, paraclinical tests, laboratory tests, transportation - ambulances, multidisciplinary care, mobility aids and home furnishings, professional care services at home and informal care, …) and questions to determine indirect direct costs (employment absenteeism, temporary or permanent reduction of working time or income, early retirement due to disability).

### Procedure

The data were collected during face-to-face interviews of 45 to 60 minutes. First the patient responded alone to three self-administered questionnaires. Then the investigator completed a medico-economic questionnaire with the patient. Depending on the type of information, the patient was asked about the period from the 3 to 12 last months.

### Data analysis

The data were analysed using SPSS version 18.0. Descriptive statistics are reported for the whole cohort and split into 3 groups according to EDSS level. Definitions of the 3 groups were decided before analysis. Ability to walk without limits defined the first group (EDSS < 4). Absence (EDSS between 4 and 5.5) or requirement of assistance (EDSS ≥ 6) to walk a limited distance defined the other two groups. Comparisons between groups were analysed using the Conover free distribution (one-factor ANOVA on ranks). When latter test showed significant group differences, post-hoc tests were then performed. The level of significance of comparisons was set at p = 0.05.

Concerning economic burden analysis, a societal perspective was adopted, in which all costs were considered without regard to the entity that pays. Costs were calculated as mean annual costs per patient. For direct costs, monetary values per unit were obtained from official sources in French administration: hospital activity tariffs (T2A), national health insurance tariffs and national price list for drugs ( http://www.ameli.fr). Indirect costs for lost productivity were evaluated by a method named "human capital approach", where the production of a person is valued at the market price (in this case, the sex-specific average salary including employers’ costs) using national labour statistics ( http://www.insee.fr). For short-term sick leave, labour costs were adjusted to patients’ working hours, whereas for long-term sick leave and early retirement due to MS, the national average annual working time by sex was used. Informal care was estimated by the replacement method, where the care would be provided by a professional (driver, housekeeper, babysitter…) rather than a family member.

Correlations were explored with Spearman coefficient(r) and were considered significant with an alpha level of 0.01.

Post-hoc analysis on employment status included comparisons with Wilcoxon-Mann–Whitney test between participants who have a job and those who had not.

### Level of evidence

The report provides Class IV evidence and is a single observational study without controls.

## Results

Among 70 eligible patients with diagnosis of MS and treatment by natalizumab in our centre, because of time-consuming interviews and a limited time available in this preliminary study, 42 were consecutively solicited. All patients being asked to participate accepted it, except one. Demographic and disease characteristics are presented in Table 1. Sex-ratio Male/Woman was 7/34 and mean age was 42.8 (SD 9.5) [23–72] years. Disease duration was 11.7 (SD 7.4) [2–40] years. EDSS score was 4.0 (SD 1.6) [1.5-7.0]. Demographic characteristics (age, sex, disease duration and EDSS) were not significantly different between participants and patients followed in our centre and treated with natalizumab, who were not included. Significant differences between groups for demographic and clinical data, utility, social participation and economic costs are reported in Table 1. For MS-specific QOL, only aggregated scores MSQOL-P and MSQOL-M are presented.

### Social participation and autonomy

**Figure 1:**
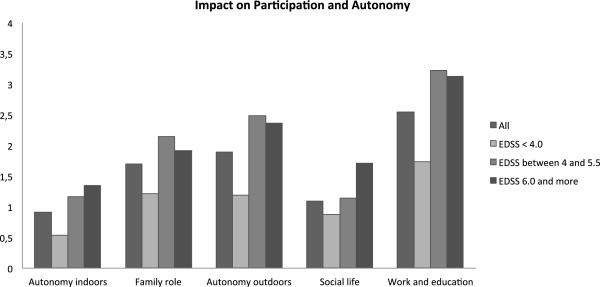
**Mean scores for each IPA domain, in the entire population and 3 groups separated by EDSS.** Scores for each domain range from 0 to 4; lower scores indicate better social participation.

Scores of the five dimensions of IPA were notably increased, with higher scores expressing greater participation reduction (Figure 1). Some domains of IPA questionnaire were moderately correlated with EDSS, MSQOL-P and MSQOL-M, and moderately to strongly with EQ5D-VAS (Table 2).

**Table 2:** Correlations (r values) of quantitative instruments (Spearman Correlation Coefficients)

	**EDSS**	**EQ5D-VAS**	**MSQOL-P**	**MSQOL-M**
IPA - Autonomy indoors	0.530**	-0.582**	-0.443*	-0.380
IPA - Family role	0.622**	-0.588**	-0.441*	-0.501**
IPA - Autonomy outdoors	0.653**	-0.724**	-0.595**	-0.640**
IPA - Social life	0.460*	-0.526**	-0.402*	-0.360
IPA - Work and education	0.534**	-0.549**	-0.388	-0.369
EDSS	NA	-0.461*	-0.255	-0.331
EQ5D-VAS	-0.461*	NA	0.678**	0.635**
MSQOL-P	-0.255	0.678**	NA	0.760**
MSQOL-M	-0.331	0.635**	0.760**	NA

**p < 0.001; *p < 0.01.

### MS specific QOL (SEP-59)

**Figure 2:**
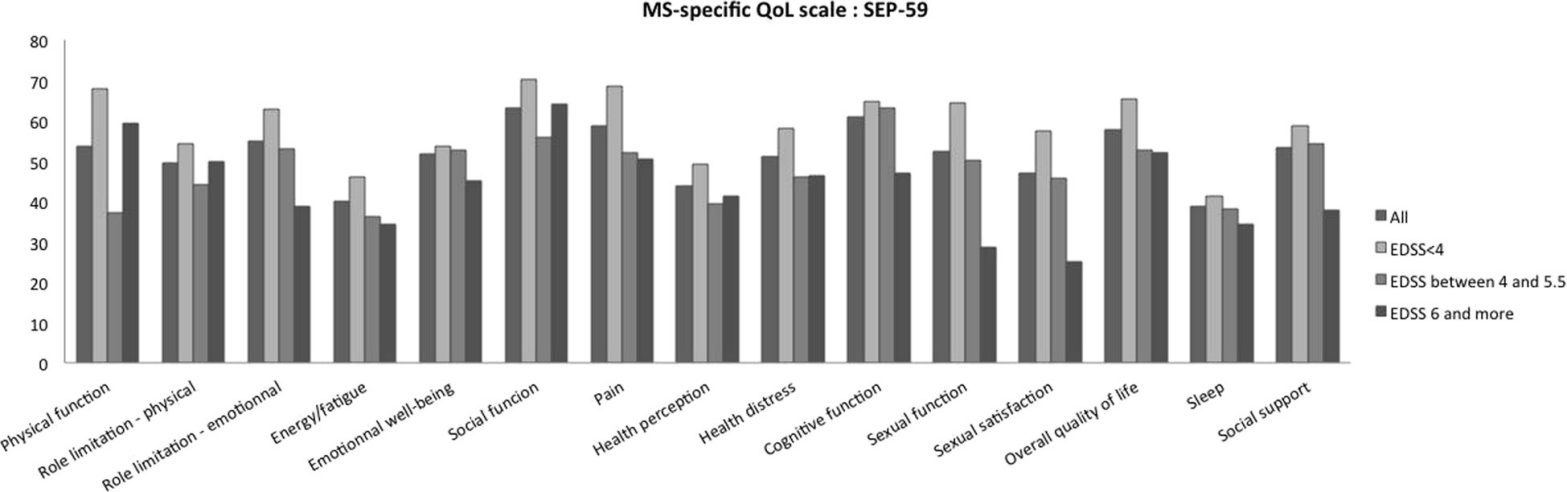
**Mean scores for each SEP-59 domain, in the entire population and 3 groups separated by EDSS.** Scores for each domain range from 0 to 100; higher scores indicate better health status, except for the pain subscale.

Mean scores of each SEP-59 domain are presented in Figure 2. Physical aggregated score MSQOL-P was significantly correlated with annual relapse rate (r = -0.435), EQ5D-VAS (r = -0.678) and IPA questionnaire’s domains (r = -0.403 to -0.595), but not with EDSS. Mental health aggregated score, MSQOL-M, was only correlated with family role (r = -0,501), autonomy outdoors (r = -0.640) and EQ5D-VAS (r = 0.635) (Table 2). Numerous SEP-59 domains were correlated with IPA subscales (data not shown). Significant correlations were found between the number of relapses during the last year and physical activities (r = -0.405), energy/fatigue (r = -0.49), pain (r = -0.415) and cognitive functions (r = -0.473). Only sexual function (r = -0.438), sexual satisfaction (r = -0.424) and overall QOL (r = -0.434) were correlated to EDSS.

### Burden of illness

The annual global cost per patient with MS was EUR68448 (SD: EUR33374) (Table 1). The largest component was the direct costs (65%). The cost attributed directly to treatment by natalizumab (the price of drug itself, one day hospitalization for administration and regular MRI survey) was estimated to EUR 30256 (44%). The annual cost excluding natalizumab was EUR38622 (SD: EUR33374). Direct non-medical cost and informal care were respectively 15.4% and 4% of total costs. Indirect costs representing lost productivity were also a substantial component (35%). Only 39% of pwMS had a full time employment. The proportion of early retirement due to MS was 40%. Mean utility in the sample was 0.52 (SD 0.28). Table 1 shows utilities according to disease severity.

Total costs were correlated with four domains of the IPA questionnaire: autonomy in doors (r = 0.519, p = 0.001), family role (r = 0.583, p < 0.001), autonomy outdoors (r = 0.616, p < 0.001), work and education (r = 0.671, p < 0.001). Concerning domains of SEP-59, total costs are correlated to energy/fatigue (r = -0.481, p = 0.001), emotional well-being (r = -0.399, p = 0.01), pain (r = 0.421, p = 0.006), health perception (r = -0.419, p = 0.006), health distress (r = -0.452, p = 0.003), overall QOL (r = -0.427, p = 0.005). Total costs are also correlated with EDSS (r = 0.628, p < 0.001) and EQ5D-VAS (r = -0.512, p = 0.001). Informal care was correlated with autonomy in doors, family role, autonomy outdoors, work and education domains of IPA questionnaire (r = 0.51 to 0.59), cognitive function in SEP-59 (r = -0.413, p = 0.007), EDSS (r = 0.447, p = 0.003) and EQ5D-VAS (r = -0.513, p = 0.001). Productivity losses were only correlated with working and education domain of IPA questionnaire (r = 0.544, p = 0.01). Utility (EQ5D-VAS) is moderately to strongly correlated with the five domains of IPA, and moderately with EDSS and SEP-59 (Table 2).

### Employment status

Post-hoc analysis showed that patients, who were still employed, had lower EDSS score (p < 0.001) and higher education level (p = 0.02) in comparison to patients without working activity. Social participation was equally better when employment was maintained for the following domains of IPA: family role (p = 0.02), autonomy outdoors (p < 0.01), social life (p = 0.01) and work and education (p < 0.001). QOL measured with EQ-5D (p < 0.01), MSQOL-P (p < 0.01) and MSQOL-M (p < 0.01) was also better in this condition. Finally, global cost and these components were all significantly lower if employment was maintained.

## Discussion

This study confirms that medico-economic costs due to MS increase with impairment measured by EDSS. Social participation evaluated by IPA questionnaire is altered in patients with MS. We found significant correlations between IPA and QOL (EQ5D-VAS or SEP-59). Association between QOL and EDSS was smaller (EQ5D-VAS) or absent. These results argue for a greater role of social participation than impairment itself (EDSS) to explain variations of QOL. Other while, moderate to strong correlations are evidenced between functional impairment (EDSS), social participation and economic costs.

Patients’ perception of social participation in our study was comparable with the results described in UK validation study of IPA [18]. The biggest change of IPA scores was present between the 2 groups with EDSS range of 0–3.5 and 4.0-5.5 for the following domains of IPA: autonomy indoors, family role, autonomy outdoors and work and education. The known shift of utility, which occurs when patients live a transition of EDSS score in the range of 1.0-3.0 to 3.5-5.5 [14,23], seems to be similar for these domains of IPA. Groups’ stratification was based on ability to walk. So walking impairment could explain a part of this shift. Outcomes, as EDSS, EQ-5D and probably IPA and MSQOL-P, are all sensitive to walking impairment. In an other hand, IPA domain on social life and relationships was significantly altered for the group of patients with EDSS ≥ 6 in comparison with patients which had unlimited walking ability. Stability of the majority of IPA domains between the two groups with higher EDSS scores is questionable and interesting. We can hypothesize with cautious that a part of the increasing economic costs with EDSS worsening can be justify by the relative preservation of social participation. Social support and informal care could help patient to maintain some activities and a relative social participation level. On the other hand, coping mechanisms can influence the individuals’ perception of their own quality or life and social participation. Acceptance of disease could participate to limit worsening of QOL or perception of participation, when disability due to MS has progressed. But a larger study is necessary to conclude on these hypotheses.

Correlations between IPA and QOL are found in this study, in particular with generic utility (EQ-5D). An other preliminary study previously showed similar associations between specific activities like physical and leisure/recreation activities and QOL in patients with MS [24]. However some domains of IPA are not or less correlated to P- and M-MSQOL. But others factors as depression, fatigue, pain, social support, living area, religiosity, which are not lightened by our data, would probably contribute to variations of MSQOL-M [25]. In this context, IPA domain on social life and relationships was expected to be more correlated to MSQOL-M, but it was not verified in our study. Kierkegaard and colleagues recently observed that some manual dexterity, cognition and walking tests could be used as predictive factors of participation limitations [4]. Previous researches with the NARCOMS registry have incriminated the earliest mobility impairment in indirect costs’ increasing, activities of daily living, socio-economic status and utility’s declining [26,27]. Indirect costs (mostly productivity losses) driven by working ability and early retirement are often considered as the most important factor to explain global cost increasing with MS progression [10,28]. In this study, the post-hoc analysis confirmed that ability to maintain employment is an important outcome and could be considered as a good marker of well-being. Economically, patients who have a job still contribute to the Gross Domestic Product of their country. So, direct costs due to MS are at least partly neutralized by their economic contribution.

The mean overall cost of MS seems increased in comparison of previous studies. This observation is explained mostly by the high cost of natalizumab. Noteworthy, this study did not aim to evaluate a cost/benefit ratio for this treatment. The calculated global burden is similar in proportion to previous French and international data [6,10,29], usually collected by postal questionnaires and therefore weakened by inherent biases. In France, Kobelt and colleagues conducted the larger costs’ evaluation in 2007. Excluding disease-modifying drugs’ cost, estimated global burden in 2007 (39353+/-39790 EUR) is close with our results (38622 +/- 33374 EUR). Utility values (EQ5D-VAS) are also very similar with a mean value to 0.52 (SD 0.29) in 2007 compared to 0.52 (SD 0.28) in this study [29].

International Conference on Disability Outcomes in MS on May 2011 underlines how wider assessments of function are potentially useful and could be acceptable by regulators [2]. Global measures of disability should be used as adjunct to or replace MS-specific outcomes depend on the purpose of the study. Regulation agencies such as FDA in USA, NICE in UK or HAS in France recommend evaluating utility by generic measures of QOL related to health status in economic analysis of health cares (such as EQ5D-VAS, SF-6D or HUI-III). These generic tools allow comparison of a disease to another to facilitate decision-making, but these do not capture several important areas for pwMS. This may lead to an underestimation of the impact of disability in patients [30]. In "cost-utility" or "cost of illness" studies, utility which is mostly expressed like Quality-adjusted life years (QALYs), is used as a comparator outcome to economic costs driven by clinical factors like relapse rates and disease progression. QALYs measures both quantity (mortality) and quality (morbidity) of life. It reflects the relative desirability or value of the health states that are described by changes in lifestyle of patient including pain, anxiety, social functioning and ability to continue activities of daily living (including leisure). In the conventional approach, ratio between global economic costs and QALY (Costs/QALY) needs to be as small as possible and first objective of a healthcare action is to maximize utility. However using QALY method is still very controversial, particularly in population concerned with disability [31]. According to theory developed by Amartya Sen and others [32,33], the level of wellbeing of a person with disabilities is less affected by changes in health status than the effect of these changes on its functionings and ultimately on their social participation [34]. Tools that explore social participation and quality of life share some common elements [35]. In the definition of QOL by the World Health Organisation, QOL can be seen as consequence of the bio-psycho-social model defined in the International Classification of Functioning, Disability and Health (ICF). So the level of social participation could influence quality of life, but it does not exactly measure the same concept. In accordance with our results, we propose that social participation measure is a complementary outcome, which highlights different concepts than QOL level.

### Limitations

Several limitations have to be reported in our study. The small size of patients’ sample decreases the strength of associations between the different elements studied and limits the generality of ours results. However, significant correlations were reported only when alpha risk was less than or equal to 0.01. On the other hand, economic costs and observed utility values are very close to those found in previous studies. Functional assessment of patients, more precisely than does EDSS, with evaluation of walking, vision, balance, cognitive functions, as well as fatigue and depression could enable better understanding of predicting factor for impaired social participation. Finally, IPA questionnaire seems to us to be a promising tool still little used today to measure social participation and disability. Numerous tools evaluating specifically functional independence and social participation are available [35], but multiplicity of tools and often time-consuming questionnaires limit their use. Only four scales are validated in French (SMAF, LIFE-H, WHODAS-II and IPA). Among these scales, only two of them, Impact on Participation and Autonomy Questionnaire (IPA) [18] and WHODAS-II [36], have been validated for MS. All questions in IPA are related to activities or participation as defined in ICF. By contrast, some items of WHODAS II are not related to these concepts of activities or social participation [35,37]. So authors chose IPA to limit confusion in interpretation of the results. Finally a consensus is needed to achieve common disability outcomes.

## Conclusion

From a small sample of pwMS, links between progression of the disease (EDSS), generic QOL, social participation and economic costs seem obvious and consistent with previous national economic data. Moderate to strong correlations of social participation with health status (EDSS), QOL, utility and economic costs encourage exploring better these links in larger cohort.

## Competing interests

The authors declare that there is no conflict of interest related with this study.

## Authors’ contributions

AK participated in the conception and the design of the study, and in the patients’ management. He performed the statistical analysis and participated in the interpretation of the data. He wrote the first draft of the manuscript. JPM participated in its conception, the analysis and interpretation of the data. MP participated in the patients’ management and interpretation of the data. PV participated in the conception of the study, the analysis and interpretation of the data. PH participated in the conception of the study, the patients’ management, the analysis and interpretation of the data. BD participated in the conception, the analysis and interpretation of the data. All authors read and approved the final manuscript.

## Pre-publication history

The pre-publication history for this paper can be accessed here:

http://www.biomedcentral.com/1471-2377/14/115/prepub

## References

[B1] Compston A Coles A Multiple sclerosis Lancet 2002 359 1221 1231 10.1016/S0140-6736(02)08220-X 11955556

[B2] Cohen JA Reingold SC Polman CH Wolinsky JS International Advisory Committee on Clinical Trials in Multiple Sclerosis Disability outcome measures in multiple sclerosis clinical trials: current status and future prospects Lancet Neurol 2012 11 467 476 10.1016/S1474-4422(12)70059-5 22516081

[B3] Pfleger CCH Flachs EM Koch-Henriksen N Social consequences of multiple sclerosis (1): early pension and temporary unemployment–a historical prospective cohort study Mult Scler 2010 16 121 126 10.1177/1352458509352196 20007430

[B4] Kierkegaard M Einarsson U Gottberg K von Koch L Holmqvist LW The relationship between walking, manual dexterity, cognition and activity/participation in persons with multiple sclerosis Mult Scler 2012 18 639 646 10.1177/1352458511426736 21982871

[B5] Clavelou P Auclair C Taithe F Gerbaud L Quality of life in multiple sclerosis: theoretical and practical aspects Rev Neurol (Paris) 2009 165 2 F115 124 19593864

[B6] Kobelt G Berg J Lindgren P Fredrikson S Jönsson B Costs and quality of life of patients with multiple sclerosis in Europe J Neurol Neurosurg Psychiatr 2006 77 918 926 10.1136/jnnp.2006.090365 16690691 PMC2077637

[B7] Polman CH O’Connor PW Havrdova E Hutchinson M Kappos L Miller DH Phillips JT Lublin FD Giovannoni G Wajgt A Toal M Lynn F Panzara MA Sandrock AW A randomized, placebo-controlled trial of natalizumab for relapsing multiple sclerosis N Engl J Med 2006 354 899 910 10.1056/NEJMoa044397 16510744

[B8] Kappos L Radue E-W O’Connor P Polman C Hohlfeld R Calabresi P Selmaj K Agoropoulou C Leyk M Zhang-Auberson L Burtin P FREEDOMS Study Group A placebo-controlled trial of oral fingolimod in relapsing multiple sclerosis N Engl J Med 2010 362 387 401 10.1056/NEJMoa0909494 20089952

[B9] Cohen JA Barkhof F Comi G Hartung H-P Khatri BO Montalban X Pelletier J Capra R Gallo P Izquierdo G Tiel-Wilck K de Vera A Jin J Stites T Wu S Aradhye S Kappos L TRANSFORMS Study Group Oral fingolimod or intramuscular interferon for relapsing multiple sclerosis N Engl J Med 2010 362 402 415 10.1056/NEJMoa0907839 20089954

[B10] Naci H Fleurence R Birt J Duhig A Economic burden of multiple sclerosis: a systematic review of the literature Pharmacoeconomics 2010 28 363 379 10.2165/11532230-000000000-00000 20402540

[B11] Manouchehrinia A Constantinescu CS Cost-effectiveness of disease-modifying therapies in multiple sclerosis Curr Neurol Neurosci Rep 2012 12 592 600 10.1007/s11910-012-0291-6 22782520

[B12] HAS Guide Choix méthodologiques pour l’évaluation économique à la HAS http://www.has-sante.fr/portail/plugins/ModuleXitiKLEE/types/FileDocument/doXiti.jsp?id=c_1120708

[B13] NICE Guide To Methods Technology Appraisal 2013 http://www.nice.org.uk/media/D45/1E/GuideToMethodsTechnologyAppraisal2013.pdf

[B14] Wundes A Brown T Bienen EJ Coleman CI Contribution of intangible costs to the economic burden of multiple sclerosis J Med Econ 2010 13 626 632 10.3111/13696998.2010.525989 20950249

[B15] Polman CH Reingold SC Banwell B Clanet M Cohen JA Filippi M Fujihara K Havrdova E Hutchinson M Kappos L Lublin FD Montalban X O’Connor P Sandberg-Wollheim M Thompson AJ Waubant E Weinshenker B Wolinsky JS Diagnostic criteria for multiple sclerosis: Revisions to the McDonald criteria Ann Neurol 2010 2011 69 292 302 10.1002/ana.22366 PMC3084507 21387374

[B16] Cardol M de Haan RJ van den Bos GA de Jong BA de Groot IJ The development of a handicap assessment questionnaire: the Impact on Participation and Autonomy (IPA) Clin Rehabil 1999 13 411 419 10.1191/026921599668601325 10498348

[B17] Vazirinejad R Lilley JM Ward CD The "Impact on Participation and Autonomy": acceptability of the English version in a multiple sclerosis outpatient setting Mult Scler 2003 9 612 615 10.1191/1352458503ms936oa 14664475

[B18] Sibley A Kersten P Ward CD White B Mehta R George S Measuring autonomy in disabled people: Validation of a new scale in a UK population Clin Rehabil 2006 20 793 803 10.1177/0269215506070808 17005503

[B19] Poulin V Desrosiers J Validation of the French translation of the Impact on Participation and Autonomy questionnaire (IPAQ) Can J Occup Ther 2010 77 159 166 10.2182/cjot.2010.77.3.5 20597376

[B20] Chevalier J de Pouvourville G Valuing EQ-5D using time trade-off in France Eur J Health Econ 2013 14 57 66 10.1007/s10198-011-0351-x 21935715

[B21] Vernay D Gerbaud L Biolay S Coste J Debourse J Aufauvre D Beneton C Colamarino R Glanddier PY Dordain G Clavelou P Quality of life and multiple sclerosis: validation of the french version of the self-questionnaire (SEP-59) Rev Neurol (Paris) 2000 156 247 263 10740096

[B22] Vickrey BG Hays RD Harooni R Myers LW Ellison GW A health-related quality of life measure for multiple sclerosis Qual Life Res 1995 4 187 206 10.1007/BF02260859 7613530

[B23] Casado V Romero L Gubieras L Alonso L Moral E Martinez-Yelamos S Martinez-Yelamos A Carmona O Arbizu T An approach to estimating the intangible costs of multiple sclerosis according to disability in Catalonia, Spain Mult Scler 2007 13 800 804 10.1177/1352458506073480 17613609

[B24] Vanner EA Block P Christodoulou CC Horowitz BP Krupp LB Pilot study exploring quality of life and barriers to leisure-time physical activity in persons with moderate to severe multiple sclerosis Disabil Health J 2008 1 58 65 10.1016/j.dhjo.2007.11.001 21122712

[B25] Yamout B Issa Z Herlopian A El Bejjani M Khalifa A Ghadieh AS Habib RH Predictors of quality of life among multiple sclerosis patients: a comprehensive analysis Eur J Neurol 2013 20 756 764 10.1111/ene.12046 23294397

[B26] Coleman CI Sidovar MF Roberts MS Kohn C Impact of mobility impairment on indirect costs and health-related quality of life in multiple sclerosis PLoS ONE 2013 8 e54756 10.1371/journal.pone.0054756 23355896 PMC3552958

[B27] Salter AR Cutter GR Tyry T Marrie RA Vollmer T Impact of loss of mobility on instrumental activities of daily living and socioeconomic status in patients with MS Curr Med Res Opin 2010 26 493 500 10.1185/03007990903500649 20014979

[B28] Orlewska E Mierzejewski P Zaborski J Kruszewska J Wicha W Fryze W Drozdowski W Skibicka I Mirowska-Guzel D Czlonkowski A Czlonkowska A A prospective study of the financial costs of multiple sclerosis at different stages of the disease Eur J Neurol 2005 12 31 39 10.1111/j.1468-1331.2004.00950.x 15613144

[B29] Kobelt G Texier-Richard B Lindgren P The long-term cost of multiple sclerosis in France and potential changes with disease-modifying interventions Mult Scler 2009 15 741 751 10.1177/1352458509102771 19383645

[B30] Kuspinar A Mayo NE Do generic utility measures capture what is important to the quality of life of people with multiple sclerosis? Health Qual Life Outcomes 2013 11 71 10.1186/1477-7525-11-71 23618072 PMC3649951

[B31] Whitehead SJ Ali S Health outcomes in economic evaluation: the QALY and utilities Br Med Bull 2010 96 5 21 10.1093/bmb/ldq033 21037243

[B32] Sen AK Commodities and Capabilities 1999 Delhi: New York: Oxford University Press

[B33] Sen A Why health equity? Health Econ 2002 11 659 666 10.1002/hec.762 12457367

[B34] Morris C Measuring participation in childhood disability: how does the capability approach improve our understanding? Dev Med Child Neurol 2009 51 92 94 19191841 10.1111/j.1469-8749.2008.03248.x

[B35] Eyssen IC Steultjens MP Dekker J Terwee CB A systematic review of instruments assessing participation: challenges in defining participation Arch Phys Med Rehabil 2011 92 983 997 10.1016/j.apmr.2011.01.006 21621675

[B36] Garin O Ayuso-Mateos JL Almansa J Nieto M Chatterji S Vilagut G Alonso J Cieza A Svetskova O Burger H Racca V Francescutti C Vieta E Kostanjsek N Raggi A Leonardi M Ferrer M MHADIE consortium Validation of the "World Health Organization Disability Assessment Schedule, WHODAS-2" in patients with chronic diseases Health Qual Life Outcomes 2010 8 51 10.1186/1477-7525-8-51 20482853 PMC2893517

[B37] Noonan VK Kopec JA Noreau L Singer J Chan A Mâsse LC Dvorak MF Comparing the content of participation instruments using the International Classification of Functioning, Disability and Health Health Qual Life Outcomes 2009 7 93 10.1186/1477-7525-7-93 19909555 PMC2785762

